# Glucagon-Like Peptide-1 (GLP-1) Receptor Agonist Treatment After Liver Transplantation: A Hypothesis-Generating Case Report

**DOI:** 10.7759/cureus.108793

**Published:** 2026-05-13

**Authors:** Nonka N Yurukova, Bissima Sultani

**Affiliations:** 1 Gastroenterology and Hepatology, University Hospital Lozenetz, Sofia, BGR; 2 Gastroenterology and Hepatology, Sofia University St. Kliment Ohridski, Sofia, BGR

**Keywords:** glucagon-like peptide-1 receptor agonists, hypothesis-generating case report, liver transplantation, metabolic dysfunction-associated steatohepatitis (mash), possible pharmacokinetic interactions

## Abstract

Glucagon-like peptide-1 receptor agonists (GLP1RAs) are widely used for the management of type 2 diabetes (T2D) and obesity, yet their safety profile in liver transplant recipients remains insufficiently characterized. We report a case of a 59-year-old liver‑retransplanted man with metabolic dysfunction-associated steatohepatitis (MASH) who developed acute disorientation and vomiting four days after initiation of dulaglutide therapy. Although a low tacrolimus trough level (2.1 µg/L) was measured upon admission to our hospital, this reflected temporary discontinuation of the drug for four days prior to sampling. Tacrolimus concentration during symptomatic episodes was not measured. Brain imaging showed cortical atrophy without evidence of posterior reversible encephalopathy syndrome (PRES) or acute ischemia. The patient had multiple risk factors for calcineurin inhibitor neurotoxicity, including hypomagnesemia (0.68 mmol/L), chronic kidney disease (glomerular filtration rate (GFR) 49 mL/min), and extensive portal-systemic shunting. Symptoms resolved after supportive therapy and adjustment of immunosuppression. This case raises the hypothesis that GLP1RAs might alter tacrolimus pharmacokinetics through delayed gastric emptying, but our case does not prove this mechanism due to the absence of contemporary tacrolimus measurement and the presence of multiple alternative explanations. We recommend enhanced monitoring when initiating GLP1RAs in transplant recipients with predisposing risk factors for neurotoxicity. Prospective pharmacokinetic studies are needed to validate this hypothesis.

## Introduction

Glucagon-like peptide-1 receptor agonists (GLP1RAs), including single and dual glucose-dependent insulinotropic polypeptide (GIP)/GLP-1 agonists, are established therapies for type 2 diabetes (T2D) and are increasingly used for weight reduction [1]. Beyond their metabolic effects, these agents demonstrate cardiovascular and renal benefits through multiple mechanisms, including stimulation of prandial insulin secretion, appetite suppression, and delayed gastric emptying [1].

Tacrolimus, a calcineurin inhibitor (CNI), is the cornerstone of immunosuppression after liver transplantation but has a narrow therapeutic index with target trough levels of 3-5 µg/L in stable recipients [2]. Neurotoxicity occurs in 10-32% of transplant recipients and can manifest even at therapeutic drug levels [3-5]. Spectrum ranges from mild symptoms (tremor, headache) to severe manifestations, including posterior reversible encephalopathy syndrome (PRES), delirium, seizures, and psychosis [3,6,7]. Risk factors for CNI neurotoxicity include hypomagnesemia, renal dysfunction, hyperbilirubinemia, history of hepatic encephalopathy, and higher donor age [4,5,8].

GLP1RAs delay gastric emptying in a dose-dependent manner, with the largest effect occurring after the first dose (a 70-90 min delay), followed by tachyphylaxis with subsequent doses [1,9,10]. Since tacrolimus is absorbed primarily in the proximal small intestine and undergoes significant first-pass metabolism via intestinal CYP3A4 and P-glycoprotein, alterations in gastric transit time could theoretically affect its bioavailability [3]. However, the clinical significance of this interaction remains unstudied.

Several studies in patients with diabetes or obesity have reported improvements in liver enzymes and hepatic steatosis with GLP1RAs, supporting interest in their potential role in metabolic dysfunction-associated steatohepatitis (MASH) [1,11,12]. Despite their expanding use, data on GLP1RAs in liver transplant recipients remain scarce. Recent systematic reviews suggest acceptable safety profiles, with no clinically significant immunosuppressant interactions requiring dose adjustment, but concerns persist regarding possible pharmacokinetic interactions and gastrointestinal side effects that may interfere with immunosuppressive medication absorption [13-16].

## Case presentation

A 59‑year‑old man presented with recurrent vomiting and episodes of disorientation. His medical history included metabolic and alcohol-associated liver disease (MetALD) leading to decompensated cirrhosis diagnosed in 2011. At University Hospital Lozenetz, Sofia, Bulgaria, the patient underwent liver transplantation in 2014. Histological examination of the explanted liver revealed low-grade multifocal hepatocellular carcinoma (pT2NxMxV1L1R0; BCLC A3). In 2018, he underwent retransplantation due to ischemic cholangiopathy with multiple strictures of the small bile ducts.

His long‑term therapy consisted of tacrolimus 1.5 mg daily, rabeprazole, ursodeoxycholic acid, febuxostat, magnesium, carvedilol, and vitamin D. Over time, he developed portal vein thrombosis, diagnosed in 2022, involving the portal vein, splenic vein, and superior mesenteric vein, with complications of portal hypertension including esophageal varices, rectal varices, splenomegaly, and ascites. He had documented portosystemic shunting, including splenorenal shunts and a recanalized umbilical vein.

Prior to dulaglutide initiation, the type 2 diabetes in the patient was managed with dietary modification and metformin hydrochloride extended-release tablets 750 mg. On August 5, 2024, dulaglutide 1.5 mg weekly was initiated for inadequate glycemic control, grade 2 obesity (BMI 36.7 kg/m²), and MASH.

Four days after the first dulaglutide dose, the patient presented to the regional emergency department with acute disorientation and recurrent vomiting. The patient had no similar episodes of confusion before August 2024 (before dulaglutide initiation). Neurological examination revealed: alert but intermittently confused and disoriented to time; no focal motor deficits; no asterixis documented; mild tremor of hands; no seizure activity observed. Brain CT on August 10, 2024, revealed cortical atrophy without evidence of ischemic stroke, intracranial mass, or posterior circulation edema characteristic of PRES. The ammonia level was not obtained.

Tacrolimus was discontinued for five days (August 11-15, 2024). Tacrolimus trough level prior to discontinuation was not measured. Based on the interim medical report, on July 2, 2024, the tacrolimus trough level was measured at 5.7 µg/L. Dose was adjusted over time from 2 mg to 1.5 mg daily, with the most recent change made in November 2023.

On August 16, 2024, tacrolimus therapy was resumed at a dose of 1.5 mg daily. On the third day after resuming tacrolimus (August 18, 2024), the patient required readmission to the Regional Hospital due to recurrent disorientation, vomiting, and transient generalized dystonia. Subsequent CT scan showed no changes compared to the previous scan. Treatment included an antibiotic (imipenem), a corticosteroid (methylprednisolone), an antiemetic (metoclopramide), and supportive care.

On August 22, 2024, the patient was transferred to University Hospital Lozenetz. On arrival, he was hemodynamically stable (BP 120/70 mmHg, HR 60 bpm) but reported intermittent confusion and disorientation. Physical examination revealed: pale mucous membranes, mild pretibial edema, and no asterixis.

Laboratory results are summarized in Table 1. Key findings at admission (August 22, 2024) included: mild renal impairment (creatinine 144 μmol/L, glomerular filtration rate (GFR) 49 mL/min), borderline hypomagnesemia (0.68 mmol/L; normal 0.7-1.0), elevated total bilirubin (31.7 μmol/L with direct fraction 14.3 μmol/L), elevated INR (1.82), and unexpectedly low tacrolimus trough level (2.1 µg/L; target 3-5 µg/L). However, this low concentration reflected the temporary discontinuation of tacrolimus prior to sampling and does not represent the level at the time of symptom onset. MELD-Na score is 13 (indicating compensated graft function without acute liver failure) [17].

**Table 1:** Laboratory results over time. GFR: glomerular filtration rate, ALAT: alanine aminotransferase, ASAT: aspartate aminotransferase, GGT: gamma-glutamyl transferase, ALP: alkaline phosphatase, CRP: C-reactive protein, INR: international normalized ratio, TCLM: tacrolimus, TCLM C0 using liquid chromatography tandem mass spectrometry (LC-MS/MS) method: tacrolimus concentration on 0-hour, MELD-Na calculated from: Cr 144, Bil 31.7, INR 1.82, Na 139. The low tacrolimus level on day one reflects four days of drug withholding (18-22 August) and does not represent the concentration during symptomatic episodes.

Variable	First day (August 22, 2024)	Third day (August 25, 2024)	One month later	Reference range
Glucose (mmol/L)	4.2	-	6.0	3.5-6.0
Bilirubin, total (μmol/L)	31.7	-	18.9	3.4-21
Bilirubin, direct (μmol/L)	14.3	-	9.9	0–8.6
Creatinine (μmol/L)	144	124	123	63-110
GFR (mL/min/1.73 m²)	49	58	59	>90
ASAT (SGOT) (U/L)	31	22	32	0-34
ALAT (SGPT) (U/L)	27	18	26	0-55
GGT (U/L)	25	18	15	0-50
ALP (U/L)	104	-	150	0-150
Magnesium (mmol/L)	0.68	-	0.8	0.7-1.0
Potassium (mmol/L)	3.5	3.6	4.1	3.5-5.5
Sodium (mmol/L)	139	137	141	
CRP (mg/L)	3.1	3.4	16	0-5
INR	1.82		1.9	0.8–1.2
TCLM C0 (µg/L)	2.1	–	2.8	3–5
MELD-Na score	13			

Brain MRI (August 23, 2024) demonstrated general cerebral atrophy with reduced gray and white matter volume, symmetrically enlarged ventricles, and nonspecific white matter gliotic foci (Fazekas grade 1) consistent with small vessel disease. Critically, there were no findings suggestive of PRES: no posterior circulation vasogenic edema, no sulcal effacement, no restricted diffusion on diffusion-weighted imaging (DWI) sequences, and no microhemorrhages on susceptibility-weighted imaging (SWI) sequences. Intracranial arteries were normal. Brain CT and MRI images are not included as findings were negative for acute pathology.

Consultation with a neurologist was conducted, who determined that there were no clinical signs of a neurological disease. Supportive therapy included intravenous fluids, electrolyte repletion (calcium gluconate, magnesium sulfate), and neuroprotective agents (piracetam 3000 mg daily). Symptoms of the patient gradually improved, and he was discharged on August 26, 2024, in stable condition. The total hospital stay is four days (August 22-26, 2024).

Discharge medications included tacrolimus 1.5 mg daily, rabeprazole 20 mg twice daily, ursodeoxycholic acid 500 mg three times daily, magnesium orotate 500 mg three times daily, vitamin D eight drops daily, carvedilol 6.25 mg twice daily, febuxostat 120 mg weekly, apixaban 2.5 mg twice daily, furosemide 20 mg daily, probiotic, and dietary modification.

One month later (September 2024), laboratory values had improved (tacrolimus 2.8 µg/L, creatinine 123 μmol/L, magnesium 0.7 mmol/L) and neurological symptoms had completely resolved.

## Discussion

This case describes a liver transplant recipient who developed acute neurological symptoms shortly after initiating dulaglutide therapy. While the temporal relationship suggests a possible drug interaction, multiple alternative explanations must be considered, given the absence of tacrolimus levels during symptomatic periods.

The clinical presentation--disorientation, vomiting, and transient dystonia--is consistent with tacrolimus-induced neurotoxicity, which occurs in 10-32% of transplant recipients [3,4]. Importantly, neurotoxicity can occur even at therapeutic tacrolimus levels, particularly in patients with predisposing risk factors [3,5]. This patient had multiple such risk factors: hypomagnesemia (0.68 mmol/L); chronic kidney disease (GFR 49 mL/min); hyperbilirubinemia (31.7 μmol/L) [3,8].

The striking temporal relationship--symptoms appearing four days after the first dulaglutide dose and recurring after tacrolimus resumption--raises the hypothesis that dulaglutide altered tacrolimus absorption. Dulaglutide delays gastric emptying by 70-90 min, with the maximal effect after the first dose due to subsequent tachyphylaxis [9,10].

Tacrolimus bioavailability depends on both intestinal absorption and first-pass metabolism via CYP3A4 and P-glycoprotein [3]. Delayed gastric emptying could theoretically prolong tacrolimus exposure to intestinal CYP3A4, potentially altering bioavailability, though the direction of effect is unpredictable. Notably, metoclopramide, which accelerates gastric emptying, is listed as potentially increasing tacrolimus levels, suggesting gastric transit time does influence tacrolimus pharmacokinetics [3].

However, this hypothesis remains unproven because: (1) No tacrolimus levels were measured during symptomatic periods. Low level (2.1 µg/L) at admission reflected drug withholding, not the concentration during acute episodes; (2) large systematic reviews of GLP1RAs in transplant recipients report no clinically significant immunosuppressant interactions requiring dose adjustment [13-16]. Singh et al. reported the largest single-center experience with dulaglutide in 63 solid organ transplant recipients, demonstrating sustained reductions in weight, BMI, and insulin requirements without increased rejection risk or need for immunosuppression adjustment [18]; (3) metabolic disturbances: hypomagnesemia alone can cause neurological symptoms independent of tacrolimus [8,19], and (4) pharmacokinetic studies of dulaglutide with other oral medications show delayed Tmax but unchanged area under the curve (AUC), suggesting total drug exposure remains stable despite delayed absorption [20]. However, these studies were conducted with drugs that have wide therapeutic indices, and the applicability to tacrolimus is uncertain [21,22]. A recent physiologically based pharmacokinetic (PBPK) modeling study demonstrated that GLP1RA-induced delays in gastrointestinal motility can substantially increase the bioavailability of coadministered oral drugs [21]. Although tacrolimus was not modeled in that study, these findings suggest that drugs with narrow therapeutic indices may be particularly susceptible to GLP1RA-mediated pharmacokinetic alterations.

Several competing etiologies warrant consideration: (1) Portal-systemic encephalopathy: the patient has extensive portal vein thrombosis with documented portosystemic shunting (splenorenal shunts, recanalized umbilical vein). A MELD-Na score of 13 indicates compensated graft function, making acute hepatic encephalopathy from liver failure less likely as the primary etiology. Studies demonstrate that noncirrhotic portal vein thrombosis causes subclinical neurological abnormalities compatible with minimal hepatic encephalopathy due to ammonia exposure from shunting. Post-transplant hepatic encephalopathy can occur with stable graft function when portal stenosis/thrombosis creates spontaneous shunts [23-26]. (2) Dehydration from gastrointestinal side effects: dulaglutide-induced nausea and vomiting could cause volume depletion, concentrating tacrolimus levels, or reducing renal clearance.

PRES occurs in 0.5-6% of solid organ transplant recipients on CNIs, typically presenting with seizures, encephalopathy, visual disturbances, and hypertension [6,7]. Characteristic MRI findings include symmetric subcortical vasogenic edema affecting parieto-occipital regions on fluid-attenuated inversion recovery (FLAIR) sequences [6,7]. This patient had none of these features: no hypertension (BP 120/70), no seizures or visual symptoms, and MRI showed only cortical atrophy without posterior circulation edema, restricted diffusion, or microhemorrhages. Therefore, PRES was appropriately excluded.

Recent evidence supports the safety of GLP1RAs in liver transplant recipients. The 2026 American Diabetes Association guidelines state that "studies have not found concern for negative interaction with immunosuppressants", though caution is advised regarding GI side effects [13]. The largest single-center study (46 liver transplant recipients) reported effective glycemic control and weight loss, with no adjustments in immunosuppression needed and only 7.7% discontinuation due to GI intolerance [16]. Singh et al. similarly reported no immunosuppression-related adverse events in 63 solid organ transplant recipients treated with dulaglutide [18].

Nevertheless, this case highlights the need for enhanced vigilance when initiating GLP1RAs in transplant recipients with multiple neurotoxicity risk factors. Practical recommendations include: correct hypomagnesemia before GLP1RA initiation (target >0.7 mmol/L) [8,19]; measure baseline tacrolimus trough levels before starting GLP1RA; recheck tacrolimus levels within three to seven days after the first GLP1RA dose, when gastric emptying delay is maximal [9,10]; consider lower starting doses (dulaglutide 0.75 mg) in high-risk patients [13]; educate patients to report neurological symptoms promptly and maintain hydration despite nausea.

Limitations

This case report has several important limitations that prevent definitive conclusions about causality: (1) Missing critical data: tacrolimus trough levels were not measured during either symptomatic episode. Low level (2.1 µg/L) documented at hospital admission reflected four days of drug withholding and does not represent the concentration at symptom onset. Without contemporary levels, we cannot confirm whether tacrolimus toxicity occurred or whether dulaglutide altered tacrolimus pharmacokinetics. (2) Incomplete diagnostic workup: ammonia level and electroencephalography were not performed, limiting our ability to exclude alternative diagnoses such as hepatic encephalopathy or metabolic disturbances. (3) Multiple confounding factors: the patient had numerous risk factors for neurological symptoms independent of any drug interaction, including hypomagnesemia (0.68 mmol/L), chronic kidney disease (GFR 49 mL/min), hyperbilirubinemia, and extensive portal-systemic shunting. (4) Single case report: as an isolated case, this report cannot establish a reproducible clinical signal or determine the incidence of potential interactions in the broader transplant population.

These limitations mean our hypothesis regarding the interaction between GLP1RA and tacrolimus remains speculative and requires validation through prospective pharmacokinetic studies with serial tacrolimus level monitoring.

## Conclusions

This case illustrates the diagnostic complexity of neurological symptoms in liver transplant recipients with multiple risk factors for encephalopathy. While the temporal relationship between dulaglutide initiation and symptom onset raises the hypothesis that GLP1RAs might alter tacrolimus pharmacokinetics through delayed gastric emptying, our case does not prove this mechanism due to the absence of tacrolimus measurements during symptomatic periods and the presence of multiple alternative explanations, including hypomagnesemia and portal-systemic shunting. The weight of published evidence from systematic reviews involving hundreds of transplant recipients suggests GLP1RAs are generally safe with no clinically significant immunosuppressant interactions. However, recent PBPK modeling suggests that GLP1RA-induced delays in gastrointestinal motility can substantially alter the bioavailability of certain coadministered oral drugs, warranting further investigation for narrow therapeutic index medications such as tacrolimus. This case demonstrates the importance of enhanced monitoring when prescribing GLP1RAs to transplant recipients with predisposing risk factors for CNI neurotoxicity, particularly hypomagnesemia and renal dysfunction.

We recommend correcting electrolyte abnormalities before GLP1RA initiation, measuring tacrolimus levels within three to seven days after starting therapy, considering lower starting doses in high-risk patients, and maintaining high clinical suspicion for neurotoxicity. Prospective pharmacokinetic studies are needed to definitively determine whether GLP1RAs alter tacrolimus absorption and to identify which patients are at increased risk. The patient reported complete resolution of confusion and disorientation following discharge. At one-month follow-up, he was functioning at his baseline cognitive level.

## References

[1] Song Q, Zhang X, Liu W (2023). Bifidobacterium pseudolongum-generated acetate suppresses non-alcoholic fatty liver disease-associated hepatocellular carcinoma. J Hepatol.

[2] Charlton M, Levitsky J, Aqel B (2018). International liver transplantation society consensus statement on immunosuppression in liver transplant recipients. Transplantation.

[3] DailyMed DailyMed (2025). Tacrolimus (Prograf) Prescribing Information, U.S. Food and Drug Administration. DailyMed.

[4] Lué A, Martinez E, Navarro M (2019). Donor age predicts calcineurin inhibitor induced neurotoxicity after liver transplantation. Transplantation.

[5] Verona P, Edwards J, Hubert K (2024). Tacrolimus-induced neurotoxicity after transplant: a literature review. Drug Saf.

[6] Fugate JE, Rabinstein AA (2015). Posterior reversible encephalopathy syndrome: clinical and radiological manifestations, pathophysiology, and outstanding questions. Lancet Neurol.

[7] Fugate JE, Hawkes MA, Rabinstein AA (2025). Posterior reversible encephalopathy syndrome: evolving insights in diagnosis, management, and outcomes. Lancet Neurol.

[8] Thompson CB, June CH, Sullivan KM, Thomas ED (1984). Association between cyclosporin neurotoxicity and hypomagnesaemia. Lancet.

[9] Jalleh RJ, Plummer MP, Marathe CS (2024). Clinical consequences of delayed gastric emptying with GLP-1 receptor agonists and tirzepatide. J Clin Endocrinol Metab.

[10] Hiramoto B, McCarty TR, Lodhia NA (2024). Quantified metrics of gastric emptying delay by glucagon-like peptide-1 agonists: a systematic review and meta-analysis with insights for periprocedural management. Am J Gastroenterol.

[11] Armstrong MJ, Gaunt P, Aithal GP (2016). Liraglutide safety and efficacy in patients with non-alcoholic steatohepatitis (LEAN): a multicentre, double-blind, randomised, placebo-controlled phase 2 study. Lancet.

[12] Sanyal AJ, Newsome PN, Kliers I (2025). Phase 3 trial of semaglutide in metabolic dysfunction-associated steatohepatitis. N Engl J Med.

[13] American Diabetes Association Professional Practice Committee (2026). 9. Pharmacologic approaches to glycemic treatment: standards of care in diabetes-2026. Diabetes Care.

[14] Garlatti Costa E, Bitetto D, Fornasiere E, Fumolo E, Ferrarese A, Toniutto P (2025). Efficacy and safety of GLP-1 receptor agonists and SGLT-2 inhibitors in the treatment of diabetes mellitus and obesity in liver transplant recipients: a systematic review. J Clin Med.

[15] Grancini V, Cogliati I, Alicandro G (2025). Glucagon-like peptide-1 receptor agonists in liver transplant recipients with diabetes: changes in glucose control and cardiometabolic risk factors. Front Endocrinol (Lausanne).

[16] Zheng K, Azhie A, You X (2024). Glucagon-like peptide-1 receptor agonists and sodium-glucose cotransporter-2 inhibitors for the treatment of diabetes mellitus in liver transplant recipients. Diabetes Obes Metab.

[17] Kim WR, Biggins SW, Kremers WK (2008). Hyponatremia and mortality in patients on the liver transplant waiting list. N Engl J Med.

[18] Singh P, Pesavento TE, Washburn K, Walsh D, Meng S (2019). Largest single-centre experience of dulaglutide for management of diabetes mellitus in solid organ transplant recipients. Diabetes Obes Metab.

[19] Touyz RM, de Baaij JH, Hoenderop JG (2024). Magnesium disorders. N Engl J Med.

[20] de la Peña A, Cui X, Geiser J, Loghin C (2017). No dose adjustment is recommended for digoxin, warfarin, atorvastatin or a combination oral contraceptive when coadministered with dulaglutide. Clin Pharmacokinet.

[21] Hooper L, Liu S, Pai MP (2025). GLP-1RA-induced delays in gastrointestinal motility: predicted effects on coadministered drug absorption by PBPK analysis. Pharmacotherapy.

[22] Calvarysky B, Dotan I, Shepshelovich D, Leader A, Cohen TD (2024). Drug-drug interactions between glucagon-like peptide 1 receptor agonists and oral medications: a systematic review. Drug Saf.

[23] Vilstrup H, Amodio P, Bajaj J (2014). Hepatic encephalopathy in chronic liver disease: 2014 Practice Guideline by the American Association for the Study of Liver Diseases and the European Association for the Study of the Liver. Hepatology.

[24] Ginès P, Krag A, Abraldes JG (2021). Liver cirrhosis. Lancet.

[25] Braun MM, Bar-Nathan N, Shaharabani E (2009). Postshunt hepatic encephalopathy in liver transplant recipients. Transplantation.

[26] Arab JP, Meneses L, Pérez RM, Arrese M, Benítez C (2013). Hepatic encephalopathy in a liver transplant recipient with stable liver function. Hepatology.

